# Reply to L.E. Daly et al

**DOI:** 10.1200/JCO.2016.68.9364

**Published:** 2016-08-01

**Authors:** Susanne Blauwhoff-Buskermolen, Marian A.E. de van der Schueren, Jacqueline A.E. Langius, Henk M.W. Verheul

**Affiliations:** Susanne Blauwhoff-Buskermolen, VU University Medical Center, Amsterdam, the Netherlands; Marian A.E. de van der Schueren, VU University Medical Center, Amsterdam, and HAN University of Applied Sciences, Nijmegen, the Netherlands; Jacqueline A.E. Langius, VU University Medical Center, Amsterdam, and The Hague University of Applied Sciences, The Hague, the Netherlands; Henk M.W. Verheul, VU University Medical Center, Amsterdam, the Netherlands

We thank Daly et al^1^ for providing valuable comments regarding our recent paper on the prognostic role of muscle loss during anticancer treatment in patients with metastatic colorectal cancer.^2^ We have found that muscle mass decreased significantly during chemotherapy and a decrease in muscle mass was independently associated with poor survival in patients with metastatic colorectal cancer. Daly et al^1^ correctly note that we observed no associations between a low skeletal muscle index at baseline and reduced survival, in contrast to some but not all previous studies. In our article, we provide some explanations for this discrepancy, for example, the heterogeneity regarding treatment regimens and follow-up time.^2^ Daly et al add a possible explanation as there may be a possible difference in body composition reference values between a North American (Canadian) and European population. Daly et al suggest that extrapolating cutoff points from a Canadian population to a cohort of Dutch patients may have been a suboptimal approach to identify the true prevalence of low SMI and the relationship between low SMI and survival within this cohort. We acknowledge the importance of differences in body composition between countries. For example, the Dutch population, on average, is taller and the prevalence of overweight and obesity is lower compared with the Canadian population.^3,4^ Although a large percentage of the Canadian population consists of (European) immigrants,^5^ we agree that it would be better to compare our study data with normative values derived from a European, or even a Dutch, population. Although we did find a new publication with cutoff values for an Asian population,^6^ normative data for a European population are not available yet.

There are several options to consider to overcome the question of ethnic variation in body composition in the near future. Data on body composition measured with computed tomography scans from recent European studies^7-10^ could be pooled to build a database with reference values for the European population. Another approach is to derive normative values from a healthy population, which is what our group is working on at the moment. It would then be interesting to repeat the statistical analyses of our study and to investigate whether our population truly displayed a low skeletal muscle index compared with European reference values. Only then we will be able to understand why skeletal muscle index was not associated with survival in our cohort and whether this may have been caused by choosing the wrong reference group.

In the meantime, while we await reference values for different countries and/or ethnic groups, we recommend that future studies on body composition display patient characteristics with regard to ethnicity, especially when cutoff values or reference values are being used. This does not apply to Europe alone, but also to other regions across the world.
